# Supplementary material for: ECE2 regulates neurogenesis and neuronal migration during human cortical development
Source: EMBO Rep. 2020 Mar 24;21(5):e48204. doi: 10.15252/embr.201948204 (PMC7202216; doi:10.15252/embr.201948204)
Supplement: Supplementary file 1 — Expanded View Figures PDF [file EMBR-21-e48204-s001.pdf]

Expanded View Figures

Figure EV1. *In vitro* human model systems and aRG delamination at 4 dpe upon *ECE2* KD in COs.

A, B Schemes depicting the generation of NPCs and neurons in 2D (A) [31] via picking of rosettes (green arrows) and in 3D in cerebral organoids (COs, B) [32]. For abbreviations, see Materials and Methods section. Single neuroepithelial regions are marked with pink arrows and with dotted boxes in (B) (scale bar = 200 μm).

C qPCR of *in vitro*-generated iPSC-derived NPCs vs. neurons shows higher *ECE2* mRNA expression in neurons.

D RNA sequencing data for *ECE2* at different stages of neuronal differentiation from iPSCs (http://stemcell.libd.org/scb/) [40].

E Validation of microRNAs targeting *ECE2* reveals a KD efficiency of about 40–50% of control levels.

F Immunostaining of COs at 4 dpe upon *ECE2* KD shows delamination of transfected aRG, in contrast to bipolar morphology of aRG in the control condition (aRG, apical radial glia; dpe, days postelectroporation; transfected cells are shown in green, $MAP2^{+}$ neuronal processes in magenta; scale bars = 50 μm).

G IHC for cleaved caspase-3 shows no difference in cell death upon ECE2 inhibition with PHOS for 14 days in COs (scale bars = 50 μm).

Data information: (B, F, G) The ventricle-like lumen in COs is marked with V'.

© 2020 The Authors

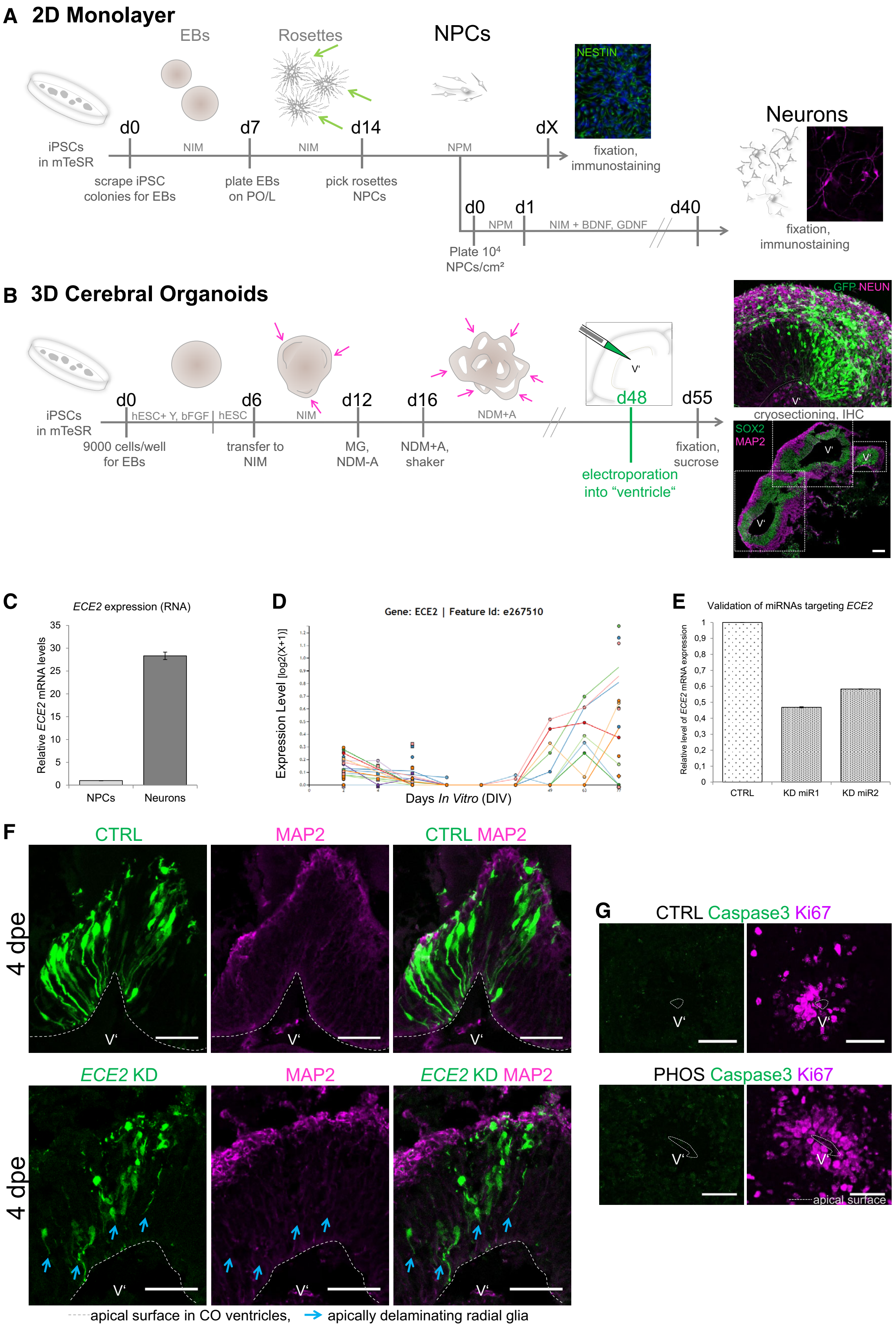

Figure EV1.

 © 2020 The Authors

Figure EV2. ***Ece2* KD *in vivo* leads to changes in apico-basal polarity and in progenitor and neuron positioning.**

A Validation of microRNA targeting *Ece2* by qPCR reveals a KD efficiency of about 65% relative to control mRNA levels.

B Binning strategy of brains at 1 dpe: Division of the thickness of the developing cortex into five equally sized bins from VZ to CP.

C–I 1 dpe upon *Ece2* KD, proliferative progenitors acquire ectopic positions (C–E). Some deep layer (G and H) and upper layer (I) neurons show the same tendency. (C) Quantification of distribution of Pax6$^+$-transfected progenitors upon *Ece2* KD relative to CTRL shows tangential shift to bin2 (n = 3 CTRL and 4 *Ece2* KD brains; P = 0.082 in one-way ANOVA; data shown as mean ± SEM). (H) Quantification of ectopic deep layer neurons at 1 dpe shows trend to ectopic, less basal position upon *Ece2* KD (n = 3 CTRL and 4 *Ece2* KD brains; P = 0.156 in one-way ANOVA; data shown as mean ± SEM). (F) aRG morphology is changed, and the apical surface shows patches lacking apically localised Arl13b at 1 dpe.

J, J' Example images at 3 dpe showing delaminated ectopic progenitors form rosettes and nodules.

Data information: (D–J) Electroporated cells shown in green; scale bar = 100 μm; ventricles marked by V.

© 2020 The Authors

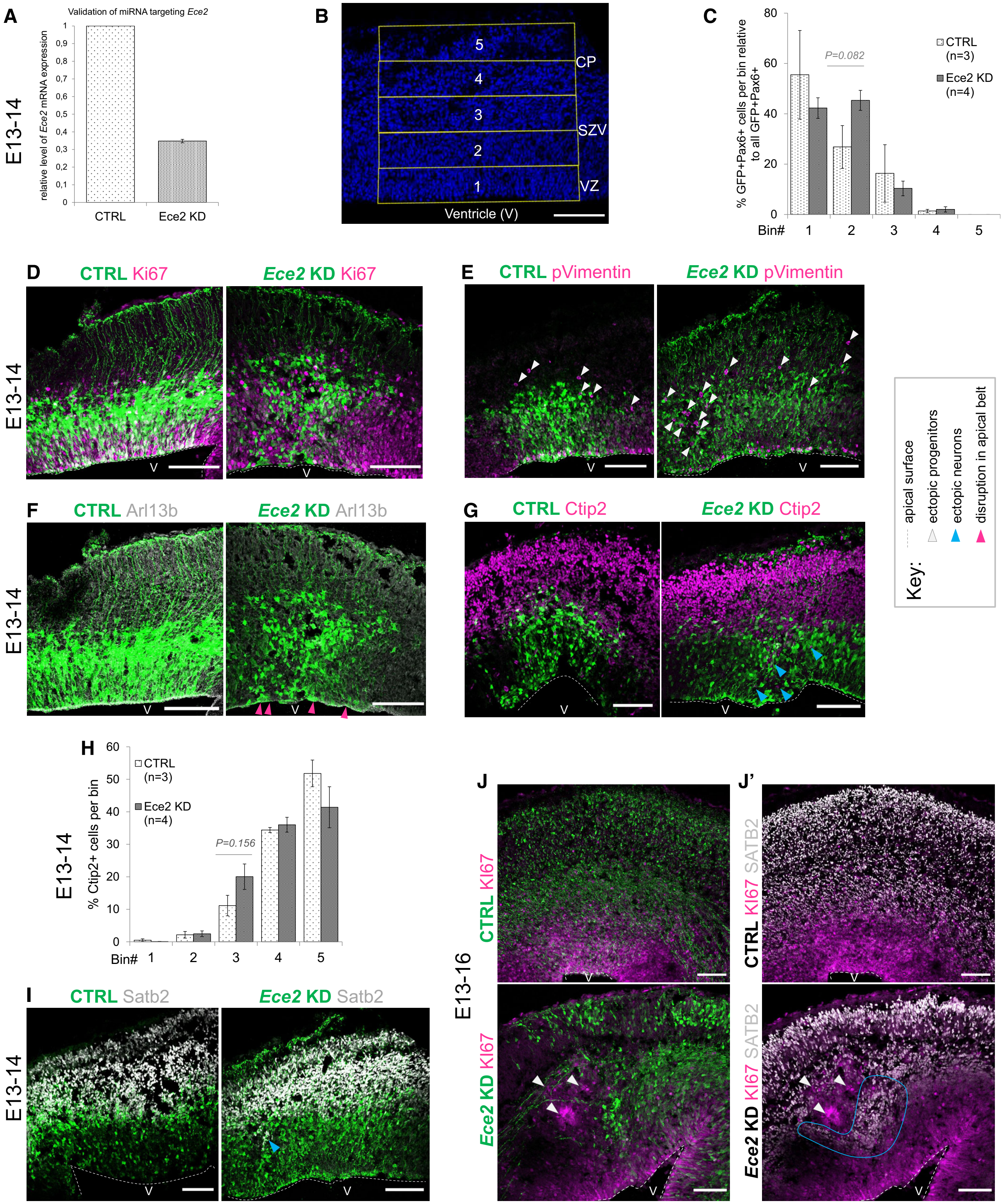

Figure EV2.

 © 2020 The Authors

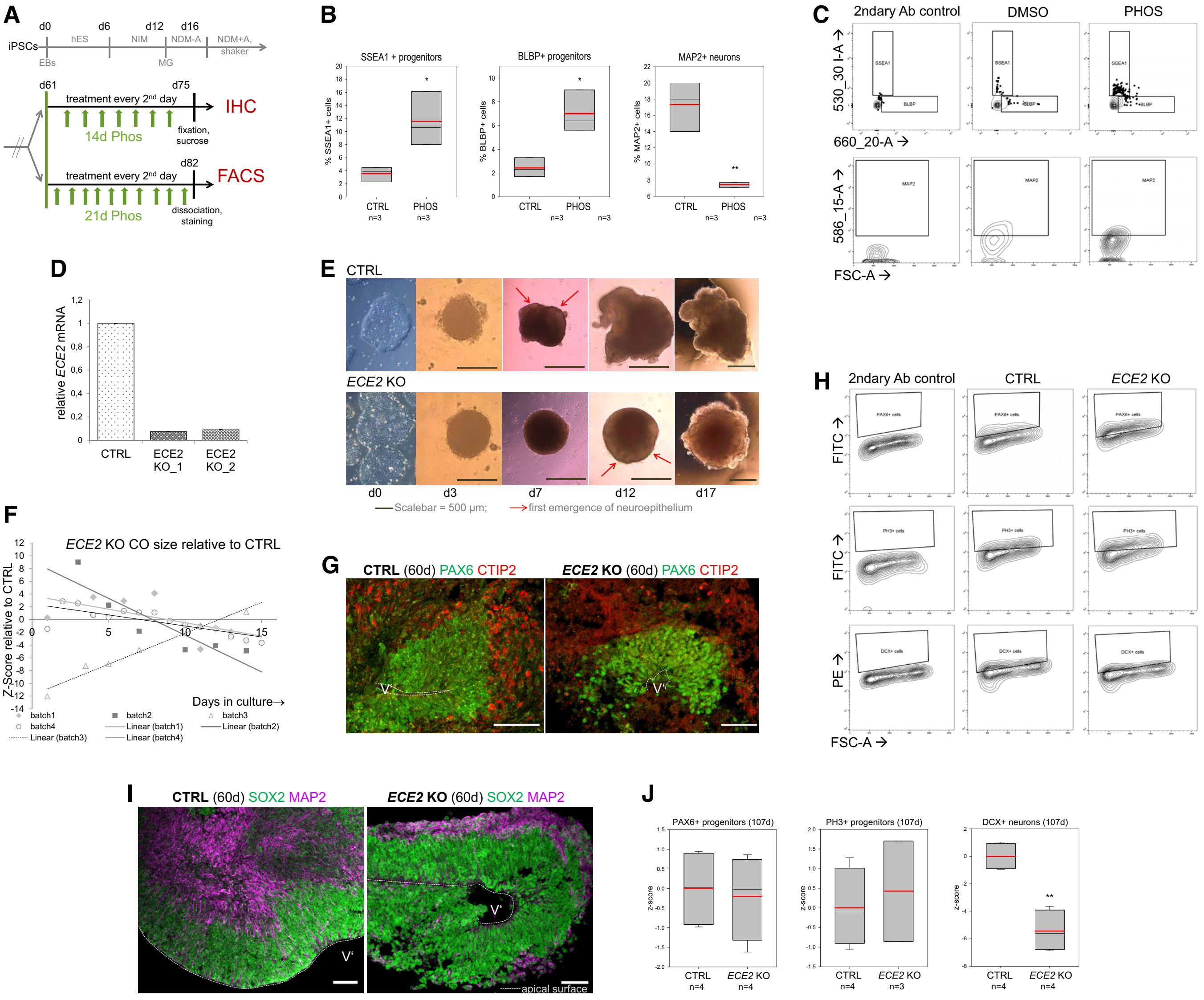

Figure EV3. ECE2 inhibition and KO cause change in cell fate in COs.

A Scheme depicting the timeline of chronic PHOS treatment in COs prior to IHC or FACS analysis (hES, NIM, NDM−/+A: media for CO generation, see Materials and Methods).

B FACS analysis of PHOS-treated COs shows an increase in neural progenitors and a decrease in neurons. Data shown as *z*-scores (n = samples of three pooled COs each; box plots: mean = red line, median = black line, box represents 25th and 75th percentiles, whiskers extend to 10th and 90th percentiles, all outliers are shown; $*P < 0.05$; $**P < 0.01$ in one-way ANOVA and Tukey's pairwise multiple comparison).

C FACS plots illustrating gating strategies for SSEA-, BLBP- and MAP2-gated cells in CTRL- and PHOS-treated COs. Gates were established using secondary antibody only as isotype control.

D qPCR confirms the absence of *ECE2* mRNA in two clones of *ECE2* KO iPSCs (data shown as mean ± SEM from triplicates).

E Brightfield (BF) images of *ECE2* KO and isogenic control iPSCs and of COs generated from them.

F Size measurement *ECE2* KO vs. CTRL COs revealed no consistent difference (data shown as *z*-scores; n = 4 batches of COs with at least 10 COs each per time point, area measured from 2D BF images in Fiji [82]).

G Example images of IHC for $PAX6^+$ dorsal neural progenitors (green) and $CTIP2^+$ deep layer neurons (red) in CTRL and *ECE2* KO COs.

H FACS plots illustrating the gating strategies for PAX6-, PH3- and MAP2-gated cells in 60-day-old CTRL and *ECE2* KO COs. Gates were established from the secondary-only control.

I Example images of progenitor zone (SOX2, green) and neuronal layer (MAP2, magenta) staining by IHC in CTRL and *ECE2* KO COs.

J FACS analysis of 107-day-old COs gated for $PAX6^+$ or $PH3^+$ progenitors or DCX^+ neurons shows no difference in progenitors, but a reduction in neurons upon *ECE2* KO also at this later stage.

Data information: Data shown as *z*-scores (n = samples of two pooled COs; box plots: mean = red line, median = black line, box represents 25th and 75th percentiles, whiskers extend to 10th and 90th percentiles, all outliers are shown; $**P < 0.01$ in one-way ANOVA and Tukey's pairwise multiple comparison). (G, I) Scale bar = 50 µm; ventricle-like lumen is marked by V'.

© 2020 The Authors

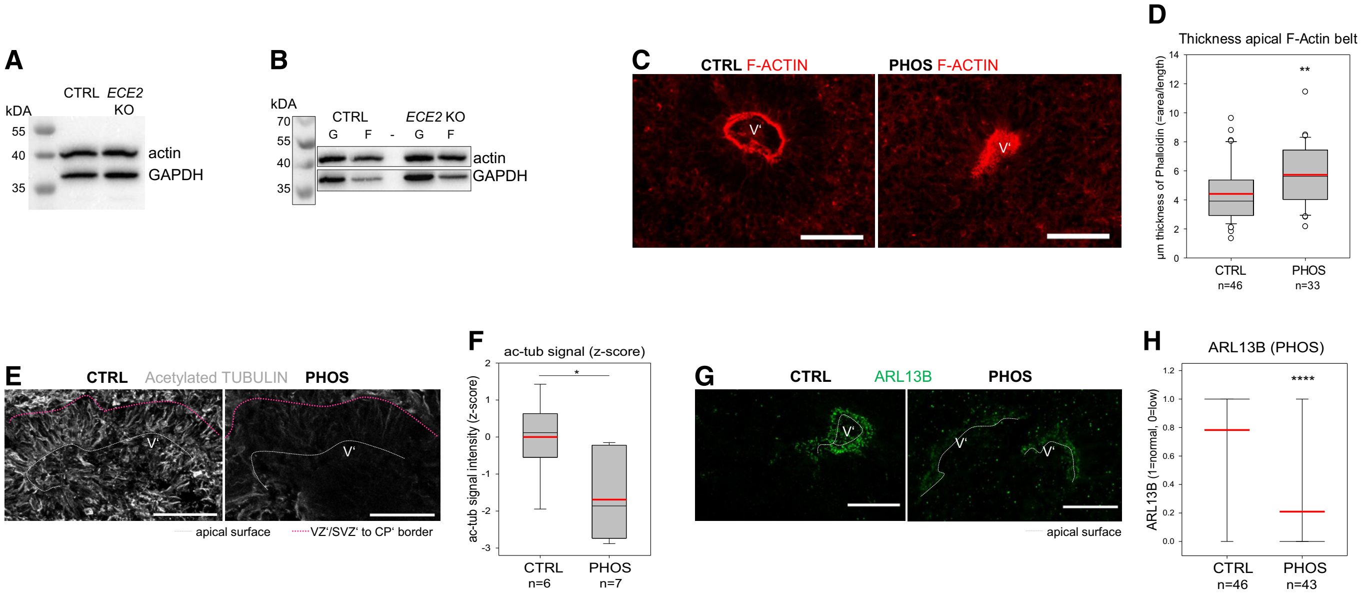

Figure EV4. ECE2 inhibition and KO cause changes in actin and microtubule cytoskeleton in COs.

A Example Western blot for total actin levels in COs with GAPDH as control.

B Example Western blot for actin levels after fractionation of G- and F-actin, using GAPDH as loading control.

C, D The thickness of the apical F-actin belt is increased in PHOS-treated COs. (C) Example images of PHOS- and CTRL-treated COs with F-actin labelled by Alexa Fluor 594-conjugated Phalloidin. (D) The thickness of the F-actin belt was assessed in Fiji [82] by measuring the area of F-actin and dividing by the length of apical surface (box plot: mean = red line, median = black line, box represents 25th and 75th percentiles, whiskers extend to 10th and 90th percentiles, all outliers are shown; n = number of analysed ventricles in 2 batches; Kruskal–Wallis one-way ANOVA on ranks and Dunn's pariwise multiple comparison: $^{**}P$ = 0.003; $^{***}P < 0.001$).

E, F The microtubule cytoskeleton is changed upon ECE2 inhibition. (E) Example images of ac-tub IHC in CTRL- vs. PHOS-treated COs. (F) Quantification of ac-tub signal as mean grey value in neuroepithelial regions of PHOS-treated COs shows significant reduction upon ECE2 inhibition (n = number of analysed ventricles; $^{*}P$ = 0.021 in one-way ANOVA and Tukey's pairwise multiple comparison).

G, H Apico-basal polarity is impaired upon ECE2 inhibition as visible in example images of ARL13B IHC in CTRL vs. PHOS (G) as assessed by quantification of germinal zones (H) for normally high ("1") vs. reduced ("0") apical ARL13B, revealing a reduction in normal ventricles in the absence of ECE2 (n = number of analysed ventricles from two batches; $^{****}P < 0.0001$ in exact binomial test).

Data information: (C, E, G) Scale bars = 50 μm; ventricle-like lumen in COs marked by V'.

 © 2020 The Authors

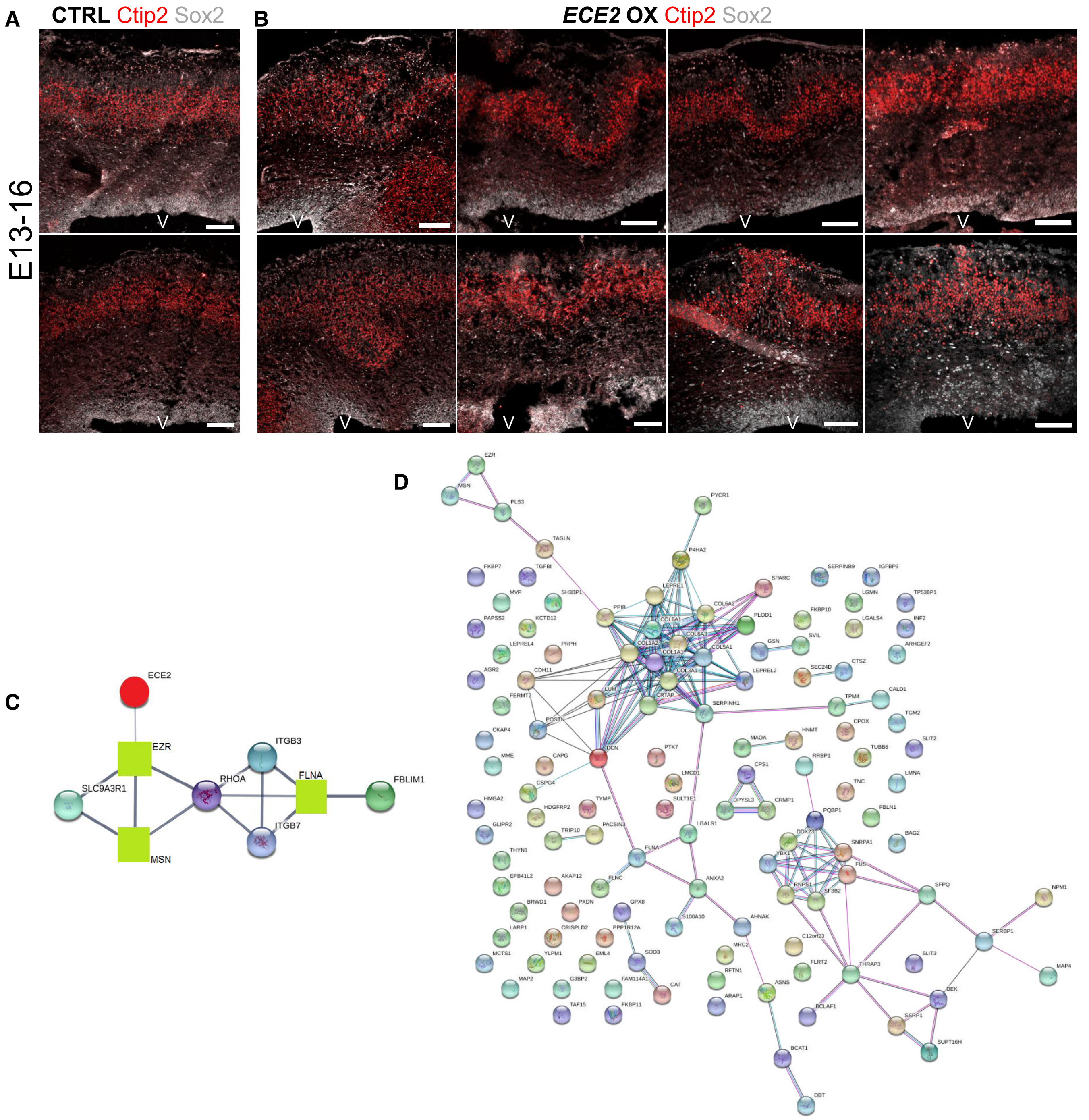

Figure EV5. Forced expression of ECE2 causes neuronal mislocalisation in the developing mouse cortex.

A, B (A) Example images of CTRL-electroporated mouse brains E13–16 and (B) example images of mouse brains at 3 dpe after forced expression of *ECE2* with IHC for Sox2^{+} neural progenitors and Ctip2^{+} deep layer neurons reveal *ECE2*'s role in neuronal positioning.

C Protein interaction analysis [52] shows network with ECE2 (red circle) and three interacting proteins that were identified as downregulated upon KO (green squares), including the known PH gene *FLNA*.

D Network analysis [52] reveals that downregulated proteins in *ECE2* KO COs are part of a tightly interconnected PPI network with ECM proteins as the biggest subnetwork.
