# Supplementary material for: ECE2 regulates neurogenesis and neuronal migration during human cortical development
Source: EMBO Rep. 2020 Mar 24;21(5):e48204. doi: 10.15252/embr.201948204 (PMC7202216; doi:10.15252/embr.201948204)
Supplement: Supplementary file 11 — Movie EV1 [file EMBR-21-e48204-s011.zip › MovieEV1/MovieEV1_legend.docx]

**Move EV1:** iDISCO whole-mount immunostained and cleared CTRL COs treated with DMSO for 2 weeks. Stainings: DAPI (blue), ARL13B (green), NEUN (red), DCX (greys). Image stack acquired at Leica SP8 Laser scanning confocal microscope; Video generated with Imaris Software.
