# Supplementary material for: ECE2 regulates neurogenesis and neuronal migration during human cortical development
Source: EMBO Rep. 2020 Mar 24;21(5):e48204. doi: 10.15252/embr.201948204 (PMC7202216; doi:10.15252/embr.201948204)
Supplement: Supplementary file 12 — Movie EV2 [file EMBR-21-e48204-s012.zip › MovieEV2/MovieEV2.docx]

**Movie EV2:** iDISCO whole-mount immunostained and cleared COs treated with PHOS for 2 weeks. This 3D view, independent of cutting angles, shows reduction in ARL13B signal compared to CTRL (EV Video 1) upon chronic ECE2 inhibition.

Stainings: DAPI (blue), ARL13B (green), NEUN (red), DCX (greys). Image stack acquired at Leica SP8 Laser scanning confocal microscope; Video generated with Imaris Software.
